# Phantom study on surgical performance in augmented reality laparoscopy

**DOI:** 10.1007/s11548-022-02809-7

**Published:** 2022-12-22

**Authors:** Christian Heiliger, Thomas Heiliger, Alessandra Deodati, Alexander Winkler, Matthias Grimm, Faisal Kalim, Javier Esteban, Lorenz Mihatsch, Lena Hiendl, Dorian Andrade, Alexander Frank, Sven Jacob, Khaled Ahmed Mohamed, Olga Solyanik, Subhamoy Mandal, Jens Werner, Ulrich Eck, Nassir Navab, Konrad Karcz

**Affiliations:** 1 Department of General, Visceral, and Transplantation Surgery, Hospital of the LMU Munich, Ludwig-Maximilians-Universität (LMU), Munich, Germany; 2 Chair for Computer Aided Medical Procedures and Augmented Reality (CAMP), Technical University of Munich (TUM), Munich, Germany; 3 Maxer Endoscopy GmbH, Wurmlingen, Germany; 4 Department of Anesthesiology and Intensive Care Medicine, Hospital of the LMU Munich, Ludwig-Maximilians-Universität (LMU), Munich, Germany; 5 Department of Radiology, Hospital of the LMU Munich, Ludwig-Maximilians-Universität (LMU), Munich, Germany; 6 School of Medical Science and Technology, Indian Institute of Technology Kharagpur, Kharagpur, India

**Keywords:** Augmented reality, Intraoperative navigation, Visualization, Laparoscopy, Phantom study, Instrument tracking

## Abstract

**Purpose:**

Only a few studies have evaluated Augmented Reality (AR) in in vivo simulations compared to traditional laparoscopy; further research is especially needed regarding the most effective AR visualization technique. This pilot study aims to determine, under controlled conditions on a 3D-printed phantom, whether an AR laparoscope improves surgical outcomes over conventional laparoscopy without augmentation.

**Methods:**

We selected six surgical residents at a similar level of training and had them perform a laparoscopic task. The participants repeated the experiment three times, using different 3D phantoms and visualizations: *Floating AR*, *Occlusion AR*, and without any AR visualization (*Control*). Surgical performance was determined using objective measurements. Subjective measures, such as task load and potential application areas, were collected with questionnaires.

**Results:**

Differences in operative time, total touching time, and SurgTLX scores showed no statistical significance ($p>0.05$). However, when assessing the invasiveness of the simulated intervention, the comparison revealed a statistically significant difference ($p=0.009$). Participants felt AR could be useful for various surgeries, especially for liver, sigmoid, and pancreatic resections (100%). Almost all participants agreed that AR could potentially lead to improved surgical parameters, such as operative time (83%), complication rate (83%), and identifying risk structures (83%).

**Conclusion:**

According to our results, AR may have great potential in visceral surgery and based on the objective measures of the study, may improve surgeons' performance in terms of an atraumatic approach. In this pilot study, participants consistently took more time to complete the task, had more contact with the vascular tree, were significantly more invasive, and scored higher on the SurgTLX survey than with AR.

**Supplementary Information:**

The online version contains supplementary material available at 10.1007/s11548-022-02809-7.

## Introduction

Minimally invasive surgery (MIS) aims to reduce the trauma of an intervention without compromising oncological outcomes [1]. Laparoscopy has become a standard procedure due to its advantages in terms of shortened recovery time, reduced postoperative pain, morbidity, wound infections, and improved cosmetic results compared to the traditional open approach [2].

In recent years, augmented reality (AR) and surgical navigation have become established in fields with minor organ deformation, such as neurosurgery and orthopedics [3–6], and have led to improved patient outcomes [4]. Implementing AR-based navigation is considerably more challenging in visceral surgery due to the large deformation of soft tissues [7].

Several detailed studies have investigated objective evaluation methods of surgical skill in open, minimally-invasive, and robotic surgery [8], but only a handful of studies have examined the impact of AR on surgical performance, and due to largely heterogeneous study setups, case series have been used more extensively. Multiple investigations suggest that AR can increase safety during surgical procedures by providing the surgeon real-time depictions of risk structures or regions of interest (ROI) [9].

Diley et al*.* [10] analyzed if AR visualizations improve surgical safety and performance by comparing different types of image guidance in a laparoscopic cholecystectomy simulator. Akladios et al. [11] evaluated the role of AR in gynecologic laparoscopic surgery in animal models. They demonstrated that navigation displayed in AR was beneficial in identifying ureters and was highly regarded among surgeons. Adballah et al*.* [12] evaluated the benefits of an AR guidance system in laparoscopic liver surgery on sheep livers and pseudo-tumors by comparing the use of Ultrasound, AR, and a combination of both. They concluded that AR or Ultrasound with AR was significantly more efficient than only Ultrasound.

Even though visualization is the primary interaction between the surgeon and the navigation system, little attention has been paid to which AR visualization technique performs best in laparoscopy. Only a few studies [10, 13] have evaluated the effect of AR visualization techniques on surgical performance and concluded that poor visualization and registration significantly reduce surgical efficiency or even harm the patient [13].

A common challenge in monitor-based AR is the correct handling of occlusions [14]. Occlusion serves as the most dominant perceptual depth cue, even higher than perspective foreshortening or binocular disparity [15, 16]. Suppose an augmented object violates the occlusion depth cue (i.e., an object below an opaque surface being visible through the surface). An observer will perceive it as floating in front of the other objects. While the observer might tolerate this mismatch to a certain degree, it is fatiguing, increases eye strain, and degrades performance [14]. This dominance of the occlusion depth cue can be an advantage if the AR visualization can incorporate occlusion.

This user study examines how a monitor-based AR laparoscopic system could improve surgical performance compared to conventional laparoscopy on a rigid 3D-printed phantom. We further compare how the correct handling of occlusions between the augmented anatomy and the surgical instruments affects surgical performance.

## Materials and methods

The experimental setup consisted of the Laparoscopic Augmentation System (LAS), two laparoscopic instruments (grasping pliers and scissors) connected to an oscilloscope, two different AR visualization methods, and three different phantoms containing rigid 3D-printed vessels.

### Phantom

**Fig. 1:**
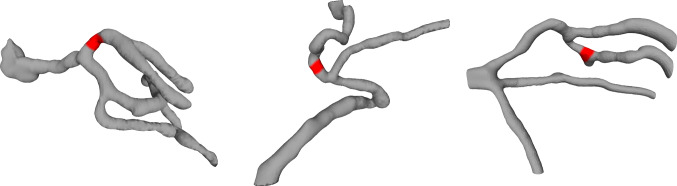
The three selected pulmonary vessels used for 3D printing. The red markings define the target regions

The study procedure required three vascular structures, as shown in Fig. 1. A radiologist segmented 12 lung CT sections. We selected three anatomical structures with similar complexity based on expert opinions from two independent surgeons.

**Fig. 2:**
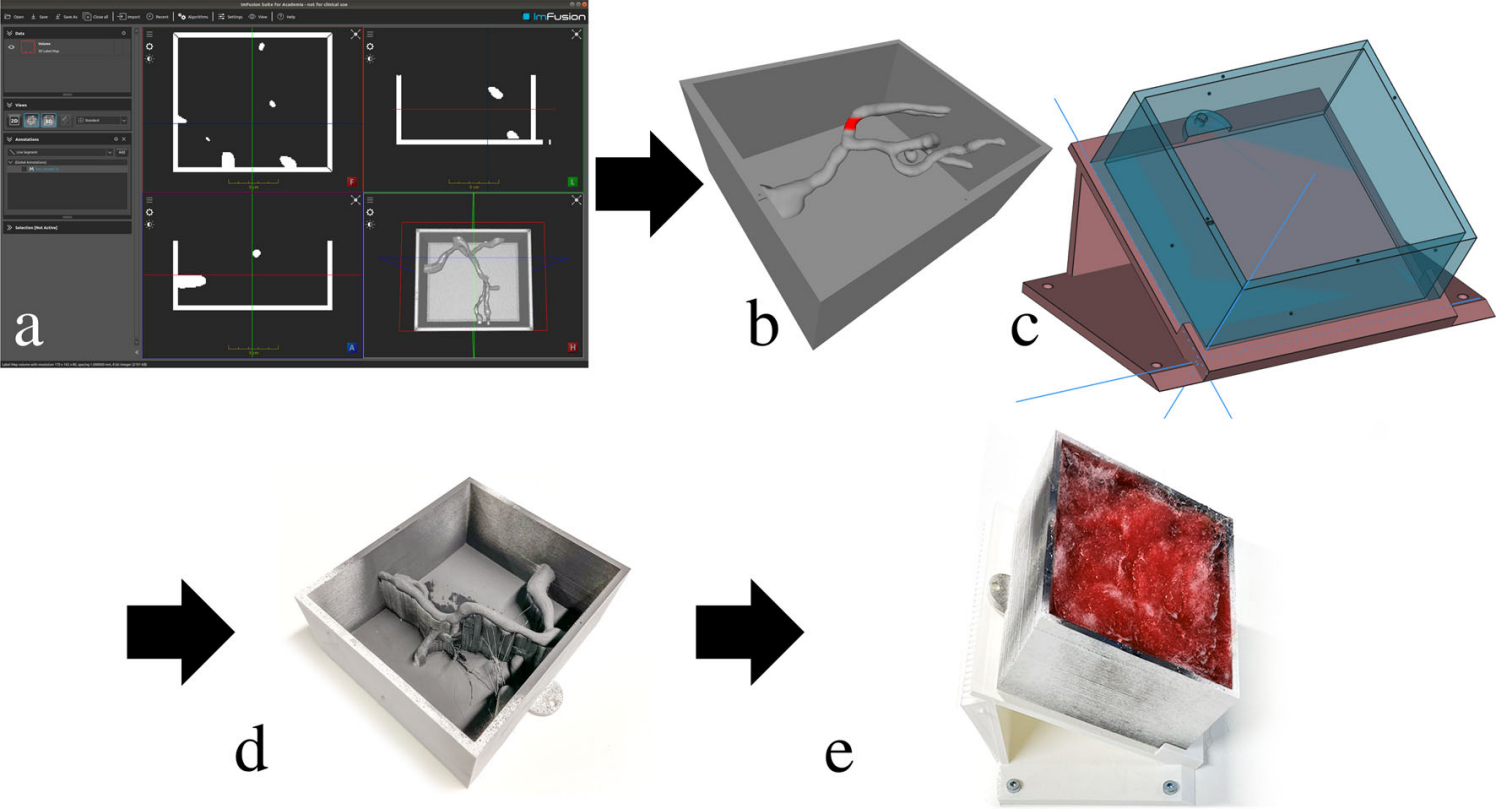
The workflow to create the phantoms: **a** we segmented blood vessels from patient CT scans in the medical image data processing suite ImFusion; **b** we embedded the 3D model of the vessel tree into a box; **c** a holder angled the box at 30°; **d** the resulting 3D-printed phantom was coated with graphite spray to provide electrical conductivity to the vascular tree; **e** we filled the phantom with cotton covered in colored wax, mimicking soft tissue

The segmentations were post-processed with a Gaussian smoothing operator in a medical image data-processing suite (ImFusion Suite, ImFusion GmbH, Germany) and arranged in a box (see Fig. 2a, b) with a 3D modeling program (Meshmixer, Autodesk Inc., USA, RRID:SCR_015736).

The box and a holder (see Fig. 2c) were 3D modeled in FreeCAD (RRID:SCR_022535) [17]. The vessel boxes were 3D-printed using selective laser sintering (SLS) in polyamide (Materialize NV, Belgium) and the holder from polylactic acid (PLA) using fused filament fabrication (FFF) (Bolt, Leapfrog 3D Printers, Netherlands). The holder angled the phantom at 30° to provide an optimal access axis for laparoscopy. An NDI infrared tracking array rigidly connected to the phantom’s base plate allowed tracking of the phantom.

**Fig. 3:**
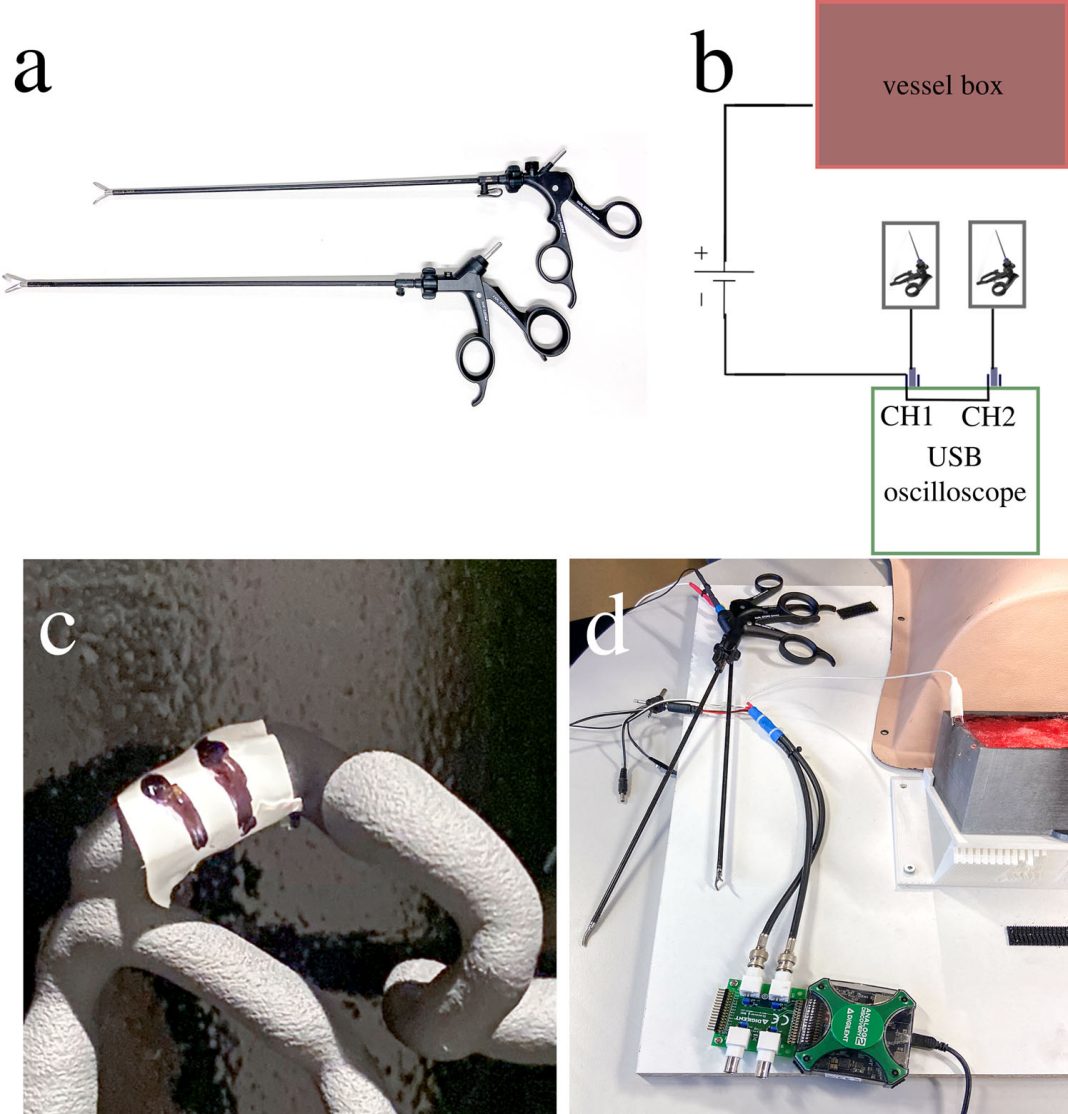
The touch measuring setup of the phantom. **a** The participants used laparoscopic pliers and scissors to accomplish the procedure. **b** Diagram of the measurement setup. **c** The participant had to identify the code on the 3D-printed model by uncovering the white insulating tape with markings. **d** Physical appearance of the measuring setup

Graphite spray on the inside of the vascular tree box provided electrical conductivity to the vascular tree **(**Fig. 2d). Electrical leads connected the vessel box to a 16 V power source and to a dual-channel USB oscilloscope (Analog Discovery 2, Digilent Inc., USA), which was, in turn, connected to the two laparoscopic instruments (Fig. 3a, b, d). This allowed measurement when each of the instruments touched the vessel. The white insulating tape with roman numerals (I, II, or III) written on it (Fig. 3c) served both as an indication of the target and as an insulator to prevent the instrument from registering contact with the vessel.

To mimic supporting and fatty tissue, we filled the physical box with absorbing cotton covered in colored gel candle wax (Exagon GmbH, Germany) (Fig. 2e), which we chose for its cost-effectiveness and satisfactory cutting sensation [18].

Finally, a hollow phantom of an abdominal torso and wall was placed over the vessel box and provided appropriate trocars to insert the laparoscopic instruments.

### Laparoscopic augmentation system (LAS)

**Fig. 4:**
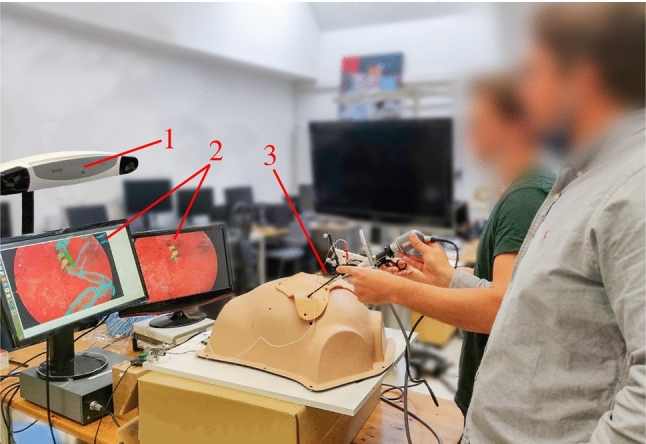
The experimental setup: **1** an NDI tracking camera, **2** two screens respectively showing the endoscopic camera feed with and without augmentation **3** a laparoscopic trainer with the endoscope and surgical instruments connected to an oscilloscope. The participant (the person in the green shirt) performed the task while instructing an assistant (the person in the gray shirt) to move the endoscope

The Laparoscopic Augmentation System (LAS) is a standard laparoscopic system with the vital addition of a computer connected to a tracking system. As shown in Fig. 4, it consists of five components: (1) a computer with a low-latency video capture card (Blackmagic DeckLink 4 K Extreme 12G), (2) an infrared-tracking system (Polaris Vega, Northern Digital Incorporated), (3) an endoscope (Viron3 HD, Maxer Endoscopy GmbH) with a passive tracking array (4) two screens, one always displaying the original laparoscopic video, the second displaying the augmented view or the original laparoscopic video in the *Control* condition, and (5) surgical instruments. The Magnum graphics middleware [19] served as the basis for the AR software, which rendered the scene in real-time and allowed for implementing advanced visualization techniques as GLSL shaders.

### AR visualization

The AR visualization consisted of overlays of vessel structures superimposed on the laparoscopic video stream in an AR monitor setup [20]. Inspired by findings for alignment in Virtual Reality [21], we opted for a representation of the virtual scene that occludes the background the least while still retaining enough information to understand the objects' shapes. Therefore, we chose a Fresnel-Derivative visualization resulting in an edge highlighting effect. Furthermore, we adapted ideas from the chroma-depth approach, which can enhance vessel perception [22]. However, instead of encoding the distance to the model as different colors, our visualization only modulated the intensity of a base color. The vessel is depicted in shades of blue, whereas the target area is in green.

**Fig. 5:**
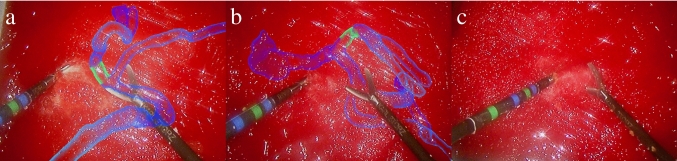
Output of the three different AR visualization conditions. **a** In *Floating AR,* occlusions of the tools with the vessels are not handled correctly, as the virtual scene will always be superimposed on top of the video stream. **b** In *Occlusion AR, *the instruments may occlude the anatomy behind them through pixel-wise classification of the video stream. **c** In the *Control* condition, participants only see the unaltered output of the laparoscopic camera without any AR

In the visualization technique, *Occlusion AR*, a random forest predictor provided pixel-wise classifications of the instruments in the live laparoscopic video stream to mask the area covered by the instrument. The predictor was trained beforehand with images from the same simulator, labeled in laparoscopic instruments and background. Morphological transformations and Gaussian blurring filtered the segmentation to achieve a gradual fade-out, and weighted averaging with the previous segmentation frames smoothed temporal inconsistencies. This effect can be seen in Fig. 5b.

In the study, each participant repeated the experiment three times, each time on a different phantom and one of the following visualization conditions:

- *Floating AR*: the AR visualization does not take occlusions of the anatomy with the instrument into account, i.e., the AR visualization always appears floating on top of the instrument (see Fig. 5a).
- *Occlusion AR*: the visualization considers the surgical instruments' segmentation, i.e., the AR anatomy is masked out at the area of the instrument. Occlusions between the anatomy and the instrument are handled, assuming the instrument always occludes blood vessels if they occupy the same image position. Therefore, the perceptual depth cue of occlusion is not violated (see Fig. 5b).
- *Control* without any AR visualization: this is the condition of using only laparoscopy as in a conventional clinical setting, without any AR visualization (see Fig. 5c).

### Study procedure

The study consisted of four phases, described as follows.

*Skill assessment survey and demographics.* Before the study, ten visceral surgery residents completed a skill assessment survey to select participants with similar levels of surgical expertise.

*Training. *After the selection, the participants proceeded with the experiment at a designated appointment. The study began with a 5-min tutorial to get comfortable with the LAS system, the instruments, and the vascular structure.

*Simulation.* Afterward, the experiment started with the laparoscopic task. The participants used laparoscopic scissors and a dissector to accomplish the procedure. An assistant, instructed with a standardized vocabulary (left, right, up, down, in, out), handled the laparoscope. The participants had two primary assignments: (1) Reaching the target by dissecting the tissue-mimicking material surrounding the vessel and (2) identifying the code, i.e., revealing the stripes on the target. Additionally, they were instructed to minimize the amount of removed tissue-mimicking material, reach the target as quickly as possible, and avoid touching the vessel. These additional requirements corresponded to the objective measures.

*Subjective Questionnaire.* The experiment ended with a survey about subjective opinions.

Half of the participants started with *Occlusion AR*, followed by *Floating AR*. For the other half, the order was flipped. The participants were unaware of the augmentation techniques. In the third run, all participants performed the *Control* condition without any AR visualization to counteract learning effects in favor of AR.

### Objective performance measures

We measured the weight of the removed material, time on task, and time touching the vessel for objective performance measures. We weighed each vessel box before and after the studies with a precision balance (440-49N, KERN & SOHN GmbH, Germany) to determine the weight of the removed material. We used the amounts of removed tissue-mimicking material to measure the simulated surgery's invasiveness. Each experiment was timed from when the participants began inserting the instruments into the laparoscopic trainer until they completed the two primary tasks. Furthermore, we recorded the time of each instrument touching the vessel using the oscilloscope by measuring the voltage at 200 Hz and registering a touch when the voltage rose above a threshold.

### Subjective measures

After each run, the participants filled out the SurgTLX survey [23]. They rated their task load on a 7-point Likert scale (ranging from 1 = “very low” to 7 = “a lot”). We evaluated the raw SurgTLX total score and the sub-scores [24].

After all three experiment runs, the participants answered a qualitative questionnaire on the usefulness of the presented AR visualization on a 4-point Likert scale, ranging from “very helpful” to “not helpful at all.” Then, the participants indicated if they would like to have any form of AR visualization for specific laparoscopic surgeries. Finally, they submitted their opinion concerning the potentially positive impact of AR on multiple surgical parameters and selected which would most benefit from AR.

### Participants

We included six participants in the study (${M}_{\mathrm{age}}=31.00, S{D}_{\mathrm{age}}=3.68$). The participants were recruited through personal contacts. 10 surgical residents were asked to self-assess their laparoscopic skills, the number of laparoscopic procedures they had performed, and state their year of training. Six surgeons with the most similar profile were selected. All participants were male. They were in the fourth, fifth, or sixth year of residency (${M}_{\mathrm{residency}}=4.83, S{D}_{\mathrm{residency}}=0.98$) and rated their laparoscopic skill on average at 2.50 ($S{D}_{\mathrm{lap}-\mathrm{skill}}=0.55$) on 5-point Likert scales (from 1 =  ``poor'' to 5 = ``very good'').

## Results

### Objective performance measures

Data analysis was performed using SPSS Statistics (IBM, USA, RRID:SCR_002865). The nonparametric Friedman test determined the significance in difference between the delta weight, total time, and time of touching among participants using AR or traditional laparoscopy. Our analysis considered values of $p <0.05$ as statistically significant.

**Fig. 6:**
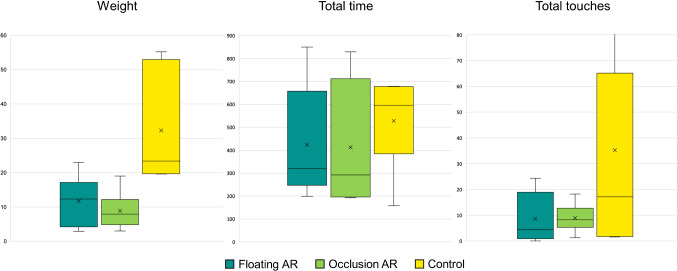
Boxplots of the objective performance measures according to the visualization mode

Regarding total execution time (see Fig. 6), the *Control* condition consistently revealed longer execution times ($M_{{\text{time,control}}} = 527.6 \,{\text{s}}, SD_{{\text{time,control}}} = 198.8\,{\text{s}}$), compared to *Occlusion AR* ($M_{{\text{time,occlusion}}} = 412.8\,{\text{ s}} , SD_{{\text{time,occlusion}}} = 270.0 \,{\text{s}}$) and *Floating AR *($M_{{\text{time,floating}}} = 424.0\,{\text{s}} , SD_{{\text{time,floating}}} = 248.3 \,{\text{s}}$). However*,* the comparison in execution time revealed that the difference is of no statistical significance ($p > 0.05$).

For touching time, the *Control* condition showed a greater time of touches to the vascular structure ($M_{{\text{touching,control}}} = 35.2\,{\text{ s}} , SD_{{\text{touching,control}}} = 49.3\,{\text{ s}}$) compared to *Occlusion AR* ($M_{{\text{touching,occlusion}}} = 8.8\,{\text{ s}} , SD_{{\text{touching,occlusion}}} = 5.6 \,{\text{s}}$) and *Floating AR* ($M_{{\text{touching,floating}}} = 8.5 \,{\text{s}} , SD_{{\text{touching,floating}}} = 9.9\,{\text{ s}}$). However, the comparison in touching time showed a difference of no statistical significance ($p > 0.05$).

When assessing the delta weight, and hence the invasiveness of the simulated intervention, the *Control* condition consistently showed a higher degree of invasiveness ($M_{{\text{weight, control}}} = 32.5 \,{\text{g}}, SD_{{\text{weight, control}}} = 16.9$) compared to *Occlusion AR* ($M_{{\text{weight,occlusion}}} = 8.9\,{\text{ g}} , SD_{{\text{weight,occlusion}}} = 5.5$) and *Floating AR* ($M_{{\text{weight,floating}}} = 11.7\,{\text{g}} , SD_{{\text{weight,floating}}} = 7.4$)*.* Friedman tests revealed a statistically significant difference ($p = 0.009$) between the three conditions. In particular, pairwise Bonferroni corrected comparisons between *Occlusion AR* and *Control* showed a statistically significant difference ($p = 0.012$). Bonferroni corrected comparisons between *Floating AR* and *Control* as well as between *Occlusion AR* and *Floating AR* showed a difference of no statistical significance ($p > 0.05$ in both cases).

### Subjective measures

**Fig. 7:**
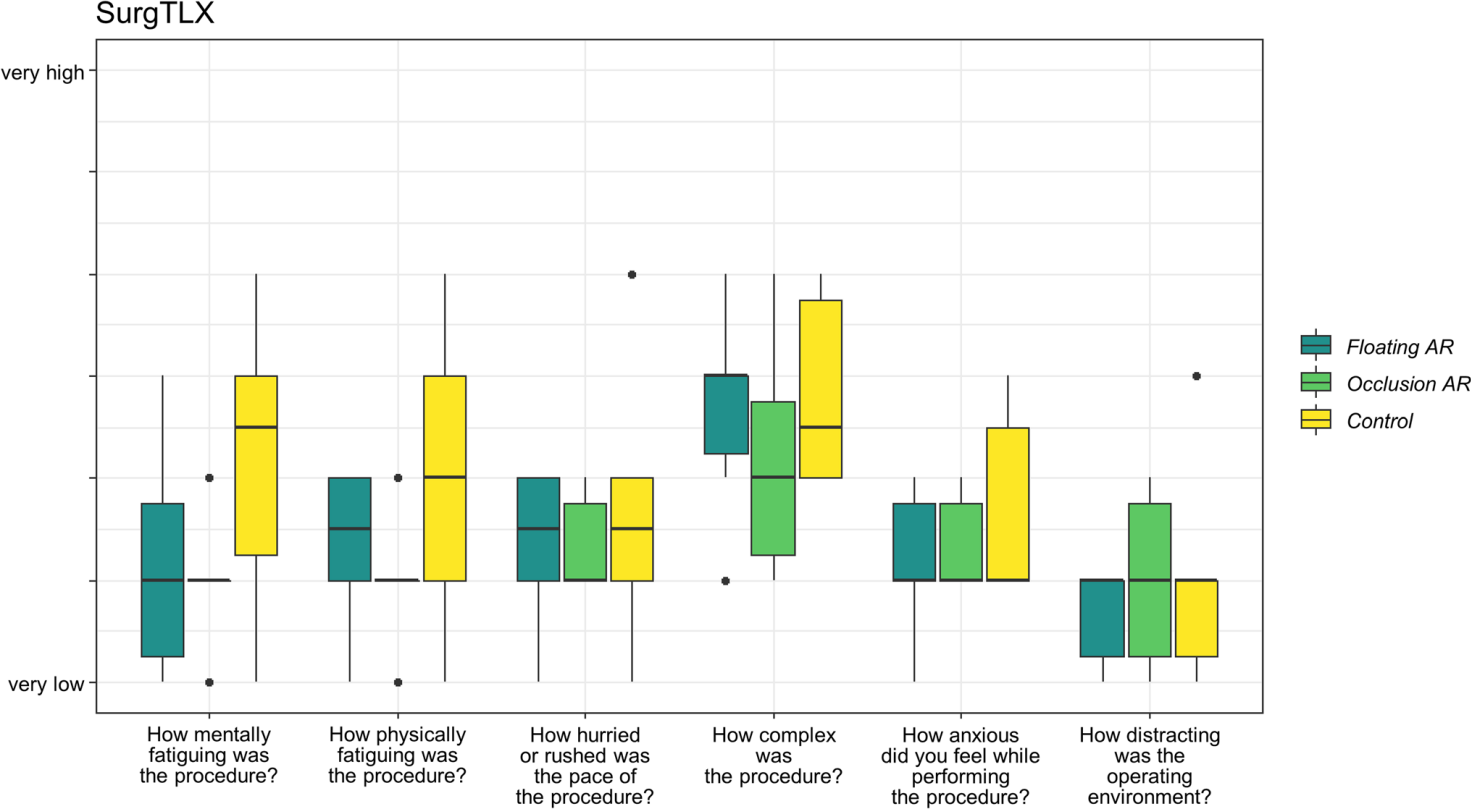
Box plots of the SurgTLX results. The participants rated their task load after each run on a 7-point Likert scale (from 1 = “very low” to 7 = “a lot”)

The results of the SurgTLX survey are depicted as box plots in Fig. 7. Our results revealed that when AR was not employed, participants consistently indicated greater task load: in the raw SurgTLX scores (taken as the mean of its sub-scores) participants assigned higher scores in the *Control* condition ($M_{{\text{rawTLX,control}}} = 2.9, SD_{{\text{rawTLX,control}}} = 1.11$), compared to the *Occlusion AR* ($M_{{\text{rawTLX,occlusion}}} = 2.3 , SD_{{\text{rawTLX,occlusion}}} = 0.24$) and *Floating AR* ($M_{{\text{rawTLX,floating}}} = 2.4 , SD_{{\text{rawTLX,floating}}} = 0.65$). This was especially evident when considering the “Mentally fatiguing” sub-score (M_TLXpℎysical,control _= 3.0, SD_TLXpℎysical,control _= 1.5;$M_{{\text{TLXmental,occlusion}}} = 2.0 , SD_{{\text{TLXmental,occlusion}}} = 0.63$; $M_{{\text{TLXphysical,floating}}} = 2.2, SD_{{\text{TLXphysical,floating}}} = 1.17$) and “Physically fatiguing” sub-score ($M_{{\text{TLXphysical,control}}} = 3.0, SD_{{\text{TLXphysical,control}}} = 1.5$;$M_{{\text{TLXphysical,occlusion}}} = 2.0,\, SD_{{\text{TLXphysical,occlusion}}} = 0.63$; $M_{{\text{TLXphysical,floating}}} = 2.3,\, SD_{{\text{TLXphysical,floating}}} = 0.8$). However, Friedman tests on the raw SurgTLX score and its sub-scores revealed differences of no statistical significance ($p > 0.05$) between the conditions.

In the further qualitative survey, more than 50% of the participants classified the LAS as beneficial for the following surgeries: liver resection (100%), sigmoid resection (100%), rectal resection (83%), left pancreatic resection (100%), gastrectomy (67%), nephrectomy (83%), cholecystectomy (67%), and right hemicolectomy (67%). Half of the participants classified the LAS as beneficial for splenectomy (50%) and TAPP (50%). Only 33% expressed a good impact of LAS on appendectomy.

All participants (100%) agreed that the LAS could positively influence specific surgical parameters. The two most commonly chosen parameters that could benefit the most from AR were “shortened operative time” (83%) and “reduction of complications” (83%). More than half of the participants stated that AR could speed up the “learning curve” (67%) and “patient safety” (67%), or lead to better “oncological outcome” (50%), and “reduce blood loss” (50%). Five out of six participants (83%) also stated that the LAS system would be very or rather helpful in identifying risk structures. The usefulness regarding stress reduction of the LAS system was rated very or somewhat helpful by 83%.

Furthermore, five of the six participants (83%) agreed that the simulation was realistic for laparoscopic interventions. Three participants (50%) indicated that the simulated tissue material needs improvement. The tracking marker on the endoscope was considered to be rarely bothersome by four individuals (67%), occasionally irritating by one (17%), and frequently irritating by one (17%). Only one participant (17%) reported noticing delays in the visualization of the LAS. Spontaneously, none of the participants addressed the difference between occlusion handling enabled or disabled. When explicitly prompted, five of the six participants (83%), however, preferred *Occlusion AR* over *Floating AR.*

## Discussion

Based on the developed AR system and the experiments performed on the phantom model, AR support for laparoscopic resection can offer advantages in terms of surgical outcomes and patient safety. In fact, the speed of the surgeon in identifying target structures was improved, and both the trauma of the surgical intervention and the risk of touching, and thereby injuring risk structures, were reduced. However, this could not be shown to be statistically significant.

Our participants, surgeons in the course of their training, believed that AR could provide significant benefits to laparoscopy, like directly guiding them but also reassuring them of their actions and ultimately increasing patient safety. Their subjective survey results also indicate a lower task load. This reinforces what current literature already suggests: AR could be a strong addition to several surgical interventions and could assist in reducing the surgeon’s stress, thus, decreasing the number of committed mistakes [25–27].

We included only surgeons of the same training level in this study to ensure the comparability of our results. We believe that AR will be of particular help to younger surgeons, as they will be the target user group. The influence of AR depending on the level of training is an exciting topic, especially concerning the development of such systems. Ideally, augmentation should also consider the user’s training level.

Our study did not measure a significant difference between the surgical performance in *Occlusion AR* and *Floating AR.* The participants were not made aware of this difference between the runs, and none explicitly mentioned noticing it. However, when asked, the participants often indicated that AR interferes with the fine preparation and that they looked at the second monitor without any AR. Our observations also suggest that most participants used the AR screen initially to understand anatomy but switched instinctively to the non-augmented screen for meticulous vessel preparation. This indicates that AR visualizations can sometimes overwhelm the surgeon with the amount of information presented. The extent to which the type of visualization influences AR performance has unfortunately received too little attention and should be further investigated [9].

### Limitations

The small sample size is an important limitation of the study. Additional research with a substantially larger number of testing participants is needed. However, a study with more participants was disproportionately time and cost-consuming because of the complex experimental setup and the length of each experiment (approx. 60 min). There were 18 vessel models in total, three for each participant. The 3D printed structures were not affected by the experiments, and the soft tissue material could be removed and the boxes refilled.

The estimation of the instrument position in the *Occlusion AR* condition was based on a pixel-wise classification of the laparoscopic video feed and, therefore, in 2D. Our random forest predictor performed well and consistently. We assumed that if the instruments were detected, they would always be in front of the anatomy. A weakness of this approach was that if the instrument was in front of the vessel, but below the soft tissue material, it could not mask the anatomy behind it, resulting in incorrect occlusion. In a follow-up study, we will incorporate 3D tracking of the instruments to ensure correct occlusion handling.

Furthermore, the phantom can be improved. Our experiment showed that laparoscopic instruments could realistically cut soft tissue, but grabbing and removing it was inconvenient in some circumstances. Participants sometimes found this frustrating since it did not reflect the actual clinical scenario. In addition, the rigidity of our model may have influenced the evaluation, as viscera and vasculature are deformable in clinical reality.

Finally, despite the above-mentioned limitations, sufficient evidence suggests that real-life clinical performance closely matches a simulated setting [28, 29]. Based on these findings, we believe that the current study is a realistic approximation of the operating room experience.

## Conclusion

In this study, we showed that participants could prepare the target structure more quickly, with less trauma and contact with risk structures with the aid of AR. In particular, a significantly lower degree of invasiveness was measured. We also saw reduced mental and physical task load in the AR conditions. Furthermore, the participating surgeons in training rated AR as very helpful, and they wished to see it incorporated into a wide range of surgical procedures. Although the results of the current study should be applied with caution to clinical practice, we believe that they accurately depict the operating room experience and that AR can have a major impact on surgery. However, further efforts to develop better technologies and evaluate their potential clinical uses are indispensable.

## Supplementary Information

Below is the link to the electronic supplementary material.

Supplementary file1 (AVI 151495 kb)

## Declarations

### Data availability statement

The data used for the current study are available from the corresponding author upon reasonable request and through collaborative investigations.
